# Assessing the impact of chronic respiratory diseases on COVID-19 in-hospital mortality in the Italian population: a comparative study

**DOI:** 10.1093/eurpub/ckaf149

**Published:** 2025-08-20

**Authors:** Silvia Fattori, Giovanna Jona Lasinio, Marco Alfò, Graziano Onder, Giada Minelli

**Affiliations:** Department of Statistical Sciences, La Sapienza University, Rome, Italy; Department of Statistical Sciences, La Sapienza University, Rome, Italy; Department of Statistical Sciences, La Sapienza University, Rome, Italy; Department of Geriatrics and Orthopaedic Sciences, Fondazione Policlinico Agostino Gemelli IRCSS, Rome, Italy; Statistical Service, Istituto Superiore di Sanità, Rome, Italy

## Abstract

The COVID-19 pandemic has severely impacted Italy, leading to millions of cases and high mortality rates. Pre-existing chronic respiratory diseases may influence patient outcomes, and understanding their role is essential for improving healthcare strategies during such crises. This study analysed data from the Italian hospital discharge records database to explore the association between chronic respiratory diseases and in-hospital mortality due to COVID-19. Patients hospitalized in 2020 were studied, with exposure to respiratory diseases assessed based on hospitalizations between 2010 and 2019. Cox regression models were used to adjust for demographic and clinical factors, including age, gender, and comorbidity. Patients with pre-existing chronic respiratory diseases (*n = *28 375, 13.9% of the total study population of 203 820) had a 71% higher risk (hazard ratio: 1.71, confidence interval: 1.54–1.90, *P* < .001) of in-hospital mortality compared to those without such conditions. Age, gender, the number of previous hospitalizations, and the Charlson comorbidity index were identified as key factors in mortality. Kaplan–Meier survival curves demonstrated significant differences in survival probabilities between exposed and unexposed groups across various age categories. Chronic respiratory diseases are associated with increased COVID-19 mortality, underscoring the need for targeted interventions in vulnerable populations to reduce the impact of future pandemics.

## Introduction

The COVID-19 pandemic has emerged as the most prominent global event in recent years, impacting countries worldwide. Italy faced significant challenges during the early stages of COVID-19, reporting ∼26 million cases and over 190 000 deaths from February 2020 [1]. To prepare for new similar challenges, it became imperative to study factors influencing patient outcomes during hospitalization to protect the most vulnerable segments of society. Early identification of risk factors predisposing individuals to unfavourable clinical outcomes in COVID-19 was crucial for early triage of patients, defining appropriate treatments (i.e. vaccination and antiviral treatment) and obtaining improved prognosis. An important predictor of the risk of progression to severe disease and death was the presence and number of pre-existing comorbidities. The presence of comorbidities in COVID-19 patients is significantly associated to adverse clinical outcomes [2–4]. Since the early stages of the pandemic, numerous studies have established a correlation between hospital admissions for COVID-19, subsequent intensive care unit admissions, and deaths, and certain pre-existing medical conditions. Cardiovascular diseases and diabetes were promptly identified as significant risk factors for severe outcomes in COVID-19 cases [5–7]. However, findings on respiratory comorbidities are less clear. A Nationwide Retrospective Cohort Study of Chinese patients provided evidence that chronic respiratory diseases were significantly associated with poor clinical outcomes of COVID-19, even after adjusting for age, sex, and other systemic comorbidities [8]. Systematic reviews and meta-analyses have also pointed out the link between chronic obstructive pulmonary disease (COPD) and COVID-19 outcomes. One study [9] considered 22 different studies mainly from China, highlighting the complications of COVID-19 in the presence of chronic respiratory diseases and/or smoking habits. It revealed a higher prevalence of respiratory diseases, particularly COPD, among severe COVID-19 patients compared to milder cases, with an odds ratio of 4.21. A separate discussion can be made regarding the presence of asthma in COVID-19 patients. A meta-analysis found no direct cause-effect relationship between asthma and COVID-19 [10]. The association with asthma with hospitalization, intensive care, and healthcare outcomes was not statistically significant. The mortality risk was found to be lower for asthmatics than for non-asthmatics. Interstitial lung disease (ILD) has emerged as another significant area of interest due to its impact on COVID-19 patients. A study revealed that for individuals with ILD, respiratory infections like COVID-19 can trigger acute exacerbations of their underlying condition, particularly in the case of idiopathic pulmonary fibrosis [11]. A more comprehensive picture, considering various respiratory diseases, emerges from a British study conducted on a cohort of over 8 million individuals between January and April 2020 [12]. The results showed that certain respiratory diseases carried higher risks of hospitalization. Specifically, lung cancer exhibited a 124% higher risk of hospitalization for COVID-19, followed by ILD (66%), idiopathic pulmonary fibrosis (59%), COPD (54%), extrinsic allergic alveolitis (35%), bronchiectasis (34%), and asthma (18%). Except for asthma, these conditions were associated with increased mortality compared to COVID-19 patients without prior respiratory diseases. In this context, our study aims to shed further light on possible risk factors influencing COVID-19 mortality. As COVID-19 primarily affects the respiratory system, this research targets patients with pre-existing respiratory diseases, to investigate whether their prior medical conditions may significantly alter the risk of in-hospital mortality due to COVID-19.

The aim of this study is to analyse the risk of mortality due to COVID-19 in patients with previous respiratory diseases using a major national data source, the hospital discharge records (HDR) database, which collects patients’ in-hospital medical histories.

## Methods

### Data source: HDR

HDR database (2010–20 period) collects data on all hospital discharge forms in the country, provided by the Italian Ministry of Health. The HDR serves as the primary instrument for collecting information on all patients discharged from both public and private hospital facilities across the entire national territory. The information collected through the HDR includes various demographic characteristics and admission-related details. Clinical characteristics are also incorporated, encompassing the main diagnosis, secondary diagnoses (up to five), as well as diagnostic or therapeutic procedures. Each patient is associated to a distinct anonymous code, facilitating the tracking of hospital admissions throughout the country over the available years. The diagnoses of patients are classified using the International Classification of Diseases, 9th revision—Clinical Modification (ICD-9-CM) coding system. For the purposes of this analysis, ordinary and day hospital admissions were considered, while admissions to long-stay care and rehabilitation facilities were excluded, as they were not pertinent to the scope of the study. To depict the hospitalization patterns of populations, the analysis focused on individuals rather than on admissions; in case multiple admissions were present for a patient, only the first COVID-19-related hospitalization in 2020 was considered.

### Study population

The study cohort selected for the present study includes only patients hospitalized in 2020 as a consequence of COVID-19 disease (ICD-9-CM codes 043, 480.4, 518.9, 519.7, 078.089). If a patient had multiple admissions for COVID-related reasons in 2020, only the initial admission was taken into consideration. Age under 14 and residence outside Italy were considered as exclusion criteria. The resulting dataset encompasses information on 203 820 patients. Exposure is defined as the presence of prior respiratory diseases; in particular, HDR from 2010 to 2019 were examined to identify admissions with at least one of the following respiratory diseases as a diagnosis:

- Pneumonia (identified by ICD-9-CM codes 480 − 486);
- COPD (codes 490−496)—bronchitis, emphysema, asthma, bronchiectasis, extrinsic allergic alveolitis, chronic airway obstructions;
- Pneumoconiosis and other lung diseases (codes 500−508)—pneumoconiosis, asbestosis, pneumonia from solids and liquids, respiratory morbidity manifestations from other and unspecified external agents;
- Other respiratory diseases (codes 510 − 519)—empyema, pleurisy, pneumothorax, abscess of the lung and mediastinum, pulmonary hypostasis congestion, post-inflammatory pulmonary fibrosis, other alveolar and parietoalveolar pneumopathies, pulmonary complications in morbid manifestations classified elsewhere, other diseases of the lung and respiratory system;
- Lung cancer (code 162).

The selected causes are the leading chronic respiratory conditions contributing to mortality in Italy [13].

The outcome of the study is in-hospital mortality, identified in the HDR by code 1 of the mode of discharge, indicating death within the hospital. Since the analysis considers hospitalizations that occurred in 2020, the death event may have occurred in 2020 or in the following months. As the hospital discharge database is not linked to the national cause-of-death registry (managed by Italian National Institute of Statistics), we only know that the patient died during hospitalization with COVID-19 as the admission diagnosis. We therefore assume that death was due to COVID-19 or its complications.

Several patient characteristics were considered for the analyses. First, we considered some demographic variables, such as gender and age class; for what concerns the latter, seven age groups were considered: 15–34, 35–44, 45–54, 55–64, 65–74, 75–84, 85+. From a clinical standpoint, we also investigated the length of stay, which was then log-transformed to achieve symmetry.

By looking at the patient’s HDR from 2010 to 2019, it was possible to calculate the Charlson comorbidity index, count the number of previous hospitalizations, and assess several individual disease indicators. The Charlson comorbidity takes into account 19 different medical conditions, with weights derived from the adjusted risk of mortality after 12 months. The total score, ranging from 0 to 37, results from summing the weights, with higher scores indicating more severe conditions [14, 15]. For the aim of the present study, Charlson comorbidity index was categorized as follows: no comorbidities (0 points), mild (1−2 points), moderate (3−4 points), or severe (>5 points) health conditions. We consider binary indicators of the following individual diseases: ischaemic hearth disease (ICD-9-CM codes 410−414), atrial fibrillation (427.3), heart failure (428), stroke (431−436), diabetes (250), dementia and Alzheimer’s disease (290, 3310), cancer (140–239), chronic liver disease and cirrhosis (571).

### Statistical methods

To analyse the evolution of survival probabilities over year 2020 and compare exposed and unexposed patients across the various age groups, we provide Kaplan–Meier survival curve estimates; the comparison between the two groups was built on using the log-rank test. Survival time was defined as the number of days from the date of hospital admission (time zero) to either in-hospital death or discharge.

A Cox proportional hazards model was employed to pursue two objectives: (ii) explore the factors that may potentially influence in-hospital mortality; (ii) estimate the impact of exposure on the outcome, while conditioning on observed confounding effects. We initially outlined a global Cox regression model (i.e. a single model fitted on the entire study population without stratifications), whose coefficients are valid for the entire country and the entire year. To account for variations due to region and pandemic waves (the threshold between the first and second wave was set to 31 July 2020), we used the region and wave IDs to define strata. Stratification allowed for the specification of a distinct baseline risk for each combination of region × wave, while covariates coefficients are assumed to be constant across strata. Analyses including stratification by pandemic wave and region are available in the Supplementary Material.

The selection of covariates for the final model was determined by constructing and comparing different models using the Akaike Information Criterion. The chosen model incorporated the following covariates: age, sex, exposure status, number of hospitalizations, Charlson index, stroke, ischaemic heart disease, dementia, diabetes, cancer, chronic liver disease, heart failure, atrial fibrillation, interaction between age and Charlson index, interaction between age and log-length of stay, and interaction between age and exposure status. As for the base levels for categorical variables, we chose the 55–64 class for the age and the male category for the gender. In the wave-specific models, Lombardy was selected as the reference region because it had the highest number of COVID-19 cases during the first wave in Italy [16]. The examination of scaled Schoenfeld residuals allowed to verify that the proportional hazards assumption was not violated.

### Ethical approval and consent to participate

This study involved the analysis of medical information obtained from the Italian HDR database. The use of these data complies with the European General Data Protection Regulation (EU GDPR 2016/679). The Italian Data Protection Authority authorized the processing of personal data relating to HDR by Italian Institute for Health and other public institutions for reasons of public interest in public health. Written consent for participation was not required for this study, in accordance with national legislation and institutional requirements.

## Results

### Descriptive analysis

The size of the exposed group is smaller than that of the unexposed: 28 375 patients (13.9% of the total) had at least one hospital admission for a respiratory disease in the past 10 years, compared to 175 445 who had not (Table 1). Focusing on the exposed group, COPDs appear in 35.5% of the previous hospitalizations, followed by pneumonia (34.2%) and other respiratory diseases (23.9%). A small proportion of patients (3.7%) had lung cancer, and an even smaller proportion (1.2%) had pneumoconiosis and other lung diseases. Finally, asthma was identified in 1.85% of the previous admissions.

**Table 1.** Study population and mortality by exposure status

	Population (*N*)	Population (%)	Deaths (*N*)	Deaths (%)
Control	175 445	86.08	37 299	21.26
Exposed	28 375	13.92	11 149	39.29
Total	203 820	100.00	48 448	23.77

**Table 2.** Distribution of demographic and clinical variables among exposed and control groups (%)

Covariate	Level	Control (%)	Exposed (%)
Gender	Male	59.02	59.85
	Female	40.98	40.15
Age group	15–34	3.93	0.76
	35–44	4.81	1.19
	45–54	11.48	3.94
	55–64	18.25	8.83
	65–74	21.44	19.38
	75–84	24.33	35.74
	>85	15.76	30.16
Charlson index	None	37.53	6.33
	Mild	47.45	38.99
	Moderate	11.65	35.47
	Severe	2.28	18.78
Diseases	Ischaemic heart diseases	12.37	30.33
	Atrial fibrillation	11.10	30.80
	Heart failure	8.13	36.76
	Stroke	8.13	18.88
	Diabetes	15.76	30.49
	Dementia	5.87	12.66
	Cancer	18.42	31.98
	Chronic liver disease	2.13	6.02

Both control and exposed groups exhibit a predominance of male patients in comparison to females (59% males–41% females for the controls, 60%–40% for the exposed). The exposed group is significantly older than the control group, with ∼65% of the patients aged 75+ and roughly 6% of patients under 54 years old.

As for clinical indicators, a very low percentage of exposed patients have a comorbidity score of 0 (because their respiratory disease is not considered in the index calculation), compared with 38% of the control group. Large differences are found in the moderate and severe categories: around 14% of the unexposed patients fall into these classes, compared to over 54% of the exposed. All considered diseases are more prevalent among the exposed, who have several additional risk factors beyond the exposure itself. Among exposed patients, the most common conditions are heart failure (36.76% vs. 8.13% in controls), cancer (31.98% vs. 18.42%), and diabetes (30.49% vs. 15.76%). Other heart-related diseases, such as atrial fibrillation (30.80% vs. 11.10%) and ischaemic heart disease (30.33% vs. 12.37%), also show notable differences. The least frequent illness in both groups is chronic liver disease (6.02% vs. 2.13%). Moreover, the exposed have an average of six previous hospitalizations in the decade 2010–9, while the unexposed group has an average of 2.

Table 2 summarizes the distribution of gender, age groups, Charlson comorbidity index scores, and selected diseases across the exposed and control groups.

Considering the length of hospitalizations, the overall average is about 14 days; there is approximately a 1-day difference between unexposed and exposed patients, namely 13.9 and 14.7 days, with the latter characterized by greater variability (SD of 14.5 vs. 12.8). For what concerns the outcome of such hospitalizations, 39.3% of exposed patients died within the hospital facility, versus 21.3% of the unexposed (Table 1). Mortality is consistently higher for the exposed; the largest differences in terms of mortality are observed in the higher age groups: between 55 and 84, the death rate of the exposed is more than 10% higher than that of the unexposed.

### Kaplan–Meier curves

**Figure 1.**
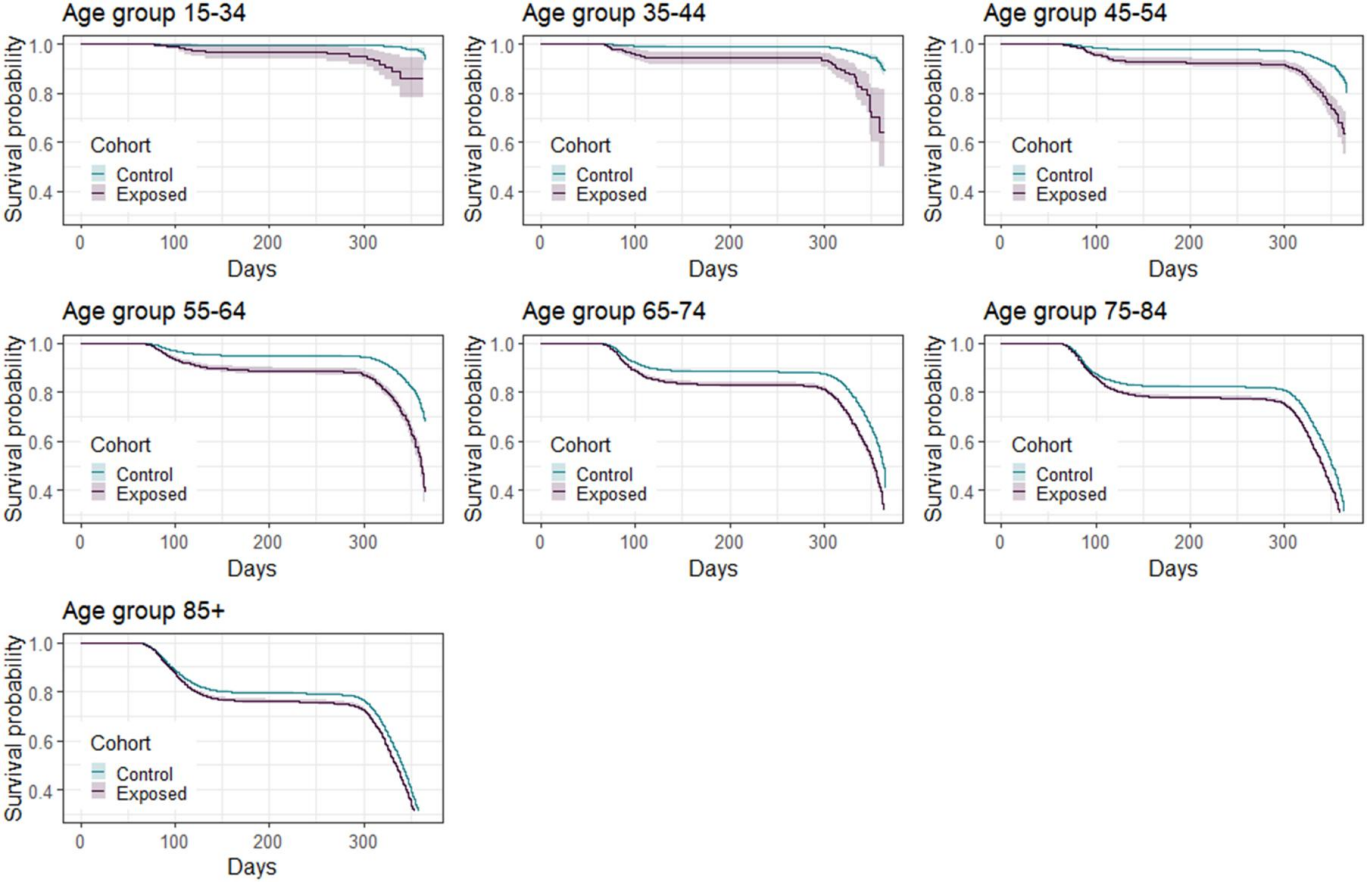
Survival curves for exposed and unexposed patients according to their age class.

As shown in Fig. 1, survival curves are quite different depending on the age class, with older individuals experiencing poorer survival outcomes. The difference between exposed and unexposed varies according to the age class: it is particularly marked among adults aged 55−64 and narrows as age increases. For all over 55s, there is a sharp decline in survival between 60 and 120 days from the beginning of 2020 (i.e. between the beginning of March and the end of April). For younger people, this decline is only observed within the exposed group: in fact, for individuals under 54 without a history of respiratory diseases, the survival probability takes on values close to 1 throughout the whole year. In the opposite situation, we find the over 85s, whose survival probability drops sharply below 80% by the end of April, both for the exposed and the unexposed. For the age group 55−64 the difference between exposed and unexposed is the largest one: here, the survival of the unexposed remains above 95%, while that of the exposed drops below 90%. Using a log-rank test, we can verify that, for every age group, the conditional (on exposure status) survival curves are significantly different (*P* < .001).

### The global Cox model

**Figure 2.**
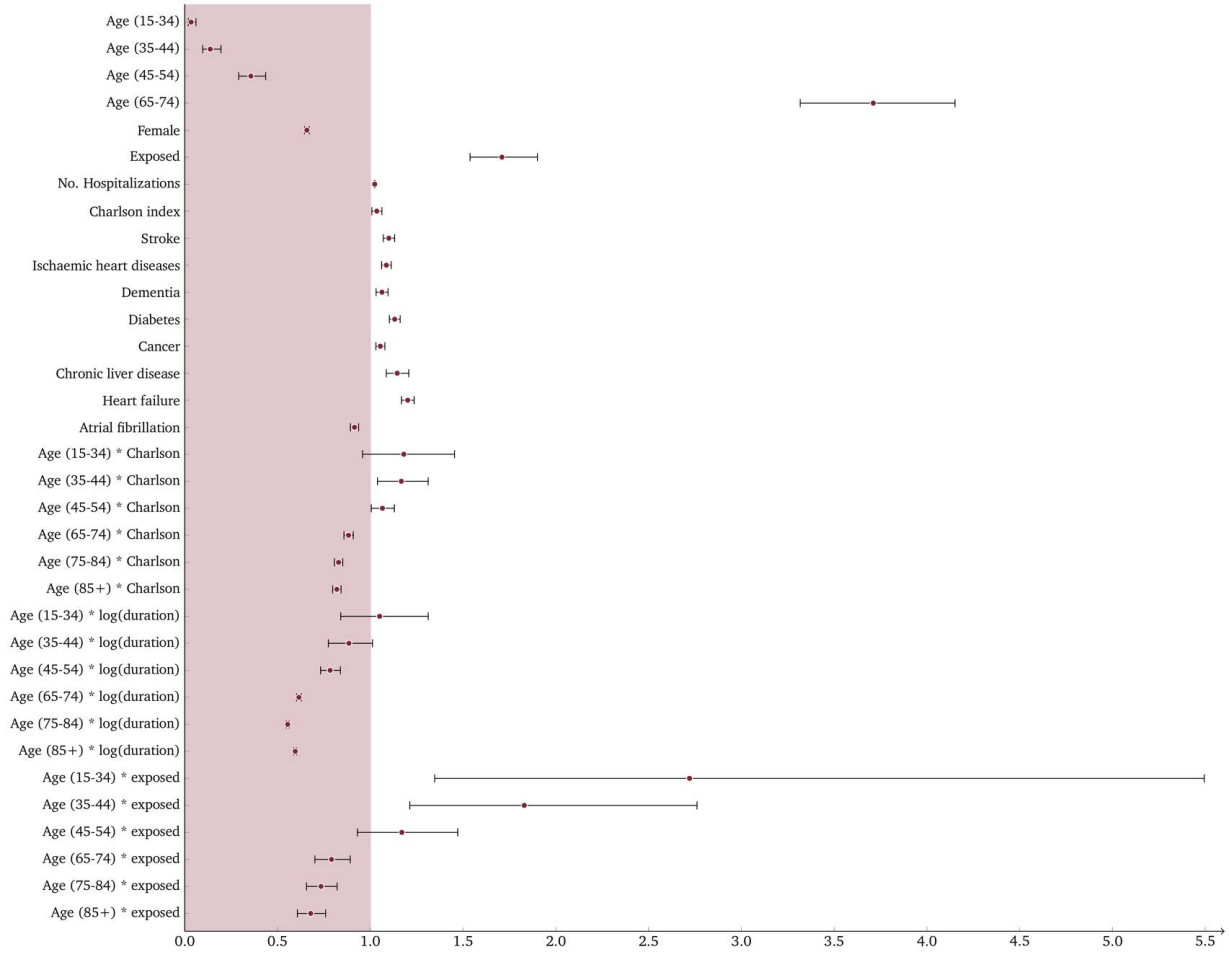
Hazard ratios and 95% confidence intervals for the covariates included in the general Cox model. The variable “duration” refers to hospital length of stay and is modelled as a time-dependent covariate.

To evaluate the effect of exposure on hospital mortality, we had to consider potential confounding factors, among observed individual features, see Fig. 2 for corresponding estimates of hazard ratios (HRs) and confidence intervals (CIs). Patients with previous respiratory disease are estimated to be associated to a 71% greater risk of mortality when all other conditions remain constant (HR: 1.71, CI: 1.54−1.90, *P* < .001). Age plays a key role in determining the mortality risk: an individual aged 15−34 has a 97% lower risk of death than the baseline individual aged 55−64 (HR: 0.035, CI: 0.03–0.04, *P* < .001); for an individual aged 35−44, this percentage is 86% (HR: 0.14, CI: 0.10–0.20, *P* < .001), and for one aged 45−54, it is 64% (HR: 0.36, CI: 0.29–0.44, *P* < .001); conversely, for older age groups, the risk of death increases exponentially. The interaction between age and exposure group provides valuable insights into how exposure alters the risk of death across different age classes. Taking the 15−34 age class as an example, we may observe that the estimated HR associated with this group is 0.035; the interaction term indicates that if a patient aged 15−34 has also had respiratory diseases, the HR is multiplied by 2.72, resulting in a value of 0.095 (HR: 2.72, CI: 1.35–5.49, *P* < .006). Similarly, exposure in the 35−44 age group exacerbates the risk of death (HR: 1.82, CI: 1.21–2.76, *P* < .004), it has no significant impact in the 45−54 class (HR: 1.17, CI: 0.93–1.47, *P* = .18), and decreases the risk for patients over 65 years old (HR: 0.79, CI: 0.70–0.89, *P* < .001). For what concerns the gender, females have an estimated 34% lower mortality risk than males (HR: 0.66, CI: 0.65–0.67, *P* < .001). Focusing on the health history of patients, each additional hospitalization in the past increases the mortality risk by 2.4% (HR: 1.02, CI: 1.02–1.03, *P* < .01), similarly each additional point in the Charlson index increases this risk by 3.5% (HR: 1.04, CI: 1.01–1.06, *P* < .001). Among individual diseases, heart failure carries the highest increased risk (20.2%; HR: 1.20, CI: 1.17–1.24, *P* < .001), followed by chronic liver disease (14.5%; HR: 1.15, CI: 1.09–1.21, *P* < .001), diabetes (13%; HR: 1.13, CI: 1.10–1.16, *P* < .001), stroke (10%; HR: 1.10, CI: 1.07–1.13, *P* < .001), ischaemic heart diseases (8.7%; HR: 1.09, CI: 1.06–1.11, *P* < .001), dementia (6.3%; HR: 1.06, CI: 1.03–1.10, *P* < .001) and cancer (5.4%; HR: 1.05, CI: 1.03–1.08, *P* < .001); conversely, patients with atrial fibrillation are found to have a 8.5% (HR: 0.92, CI: 0.89–0.94, *P* < .001) lower risk than patients with no such disease.

## Discussion

The main purpose of this research was to investigate the potential link between history of chronic respiratory disease and mortality due to COVID-19, focusing on Italian patients who were hospitalized—for the first time due to COVID-19—in 2020. To explore this association, we employed survival analysis techniques, including Kaplan–Meier estimators and Cox regression models. The aim was to evaluate the impact of previous respiratory diseases on in-hospital mortality.

The importance of conducting such a study in Italy stems from findings in multiple systematic reviews and meta-analyses revealing regional disparities in COPD and asthma prevalence among COVID-19 patients [17–19]. These differences could, however, be related to possible differential responses by regional governments and health services (which are regional in Italy), which were not considered in this study. It would be interesting in the future to investigate these regional differences more thoroughly by taking into account additional factors, such as different levels of air pollution, that may have made the general population in Northern Italy more susceptible to respiratory infections. Such variations may influence hospitalization outcomes. In general, a consistent pattern emerges, indicating a higher in-hospital mortality risk for COVID-19 patients with COPD, while no significant difference is observed for those with asthma.

It is important to note that different age groups displayed markedly distinct mortality rates, consistently showing higher rates among the exposed patients across all age categories. The Kaplan–Meier survival curves offered valuable insights into how survival probability evolves over time with significant differences between exposed and unexposed patients within each age group. These curves reveal that the most pronounced disparity in survival between exposed and unexposed patients occurs within the middle age group (55−64 years old); moreover, it is worth noting that the log-rank test confirmed statistically significant differences in survival curves between the two groups across all age categories. Another important aspect of interest is quantifying the impact of exposure on in-hospital mortality while considering potential observed confounding factors. Along with the exposure status indicator, we considered demographic (age, gender) and clinical variables (number of previous hospitalizations, Charlson comorbidity index, and specific disease indicators), as well as some interactions (such as age and Charlson index, age and exposure, and age and the logarithm of length of stay). This comprehensive approach allowed us to account for any observed individual feature that may have had a role in determining the observed outcome. Most importantly, it enabled us to estimate the effect of exposure while accounting for confounding factors. In the overall model, our analysis suggests that exposed patients have, on average, a 71% higher risk of mortality during their hospital stay compared to unexposed patients. Given the same level of exposure, the presence of additional comorbidities appears to play a significant role, particularly in younger age groups, whereas the duration of hospitalization does not seem to be influenced by patient age. The interaction between age and exposure reveals a disadvantage among younger individuals, most likely due to a more severe clinical presentation. Analyses stratified by pandemic wave showed that the risk associated with prior respiratory disease was somewhat higher during the first wave, and regional patterns of mortality differed between the two waves. A full breakdown of these results is provided in the Supplementary Material.

It is essential to note that we had to use in-hospital mortality as an endpoint as the linkage between the HDR and the living status of patients reported in the National Registry of Resident Population (Anagrafe Nazionale della Popolazione Residente) was not made available to us. However, performing a regional-level comparison using the dataset that reports COVID-19-related mortality in Italy in 2020 [20] allowed us to conclude that in-hospital mortality is a reliable proxy for the overall mortality in Italy. A limitation of this study, relying on individual patient data, is that we cannot quantify the extent to which regional health systems organization may have directly influenced in-hospital mortality.

## Conclusions

This study confirms the higher mortality risk among individuals with a pre-existing history of respiratory diseases. Patients with a previous respiratory disease exhibit a 71% higher risk of mortality when all other conditions are fixed. In this context, age plays a crucial role in determining the mortality risk, with older age groups experiencing an exponential increase in the risk of death. Multimorbidity, as measured by the Charlson index, is associated with increasing risk as the number of pre-existing pathologies increases. The findings suggest that the susceptibility to fatal outcome associated with the virus is closely linked to frailty, characterized by advanced age and a complex clinical profile.

## Supplementary Material

ckaf149_Supplementary_Data

## Data Availability

The data that support the findings of this study are available from Istituto Superiore di Sanità but restrictions apply to the availability of these data, which were used under license for the current study, and so are not publicly available. Data are however available from the authors upon reasonable request and with permission of Istituto Superiore di Sanità. Requests for access to the hospital discharge dataset should be directed at: giada.minelli@iss.it Key points Pre-existing chronic respiratory diseases increase the risk of in-hospital mortality from COVID-19 by 71%. Age, gender, number of prior hospitalizations, and the Charlson comorbidity index significantly impact COVID-19 mortality. Patients with chronic respiratory diseases show lower survival probabilities, especially in older age groups. Targeted healthcare interventions for vulnerable populations are crucial to mitigate the impact of future pandemics.
