# Supplementary material for: Assessing the impact of chronic respiratory diseases on COVID-19 in-hospital mortality in the Italian population: a comparative study
Source: Eur J Public Health. 2025 Aug 20;35(5):1058–63. doi: 10.1093/eurpub/ckaf149 (PMC12529261; doi:10.1093/eurpub/ckaf149)
Supplement: ckaf149_Supplementary_Data [file ckaf149_supplementary_data.docx]

## Supplementary material

### Cox models restricted to a pandemic wave including the regional effect

To investigate potential temporal and geographical heterogeneity in mortality outcomes, we estimated a Cox model restricted to patients hospitalized during the first wave of the pandemic (from February 1st to July 31st, 2020), explicitly including region as a covariate. The results, presented in Figure 3, show hazard ratios and 95% confidence intervals for all covariates in the model. Most Italian regions exhibited a significantly lower risk of death compared to Lombardy, which was the region most severely affected in the early months of the pandemic. In particular, residents of Lazio, Sardinia, Molise, and the Autonomous Province of Trento had an estimated risk of in-hospital death that was approximately halved relative to those in Lombardy. Conversely, no statistically significant differences were observed for Basilicata, Calabria, Marche, Sicily, and Tuscany when compared to Lombardy. It is worth noting that several Southern regions, namely Sicily, Calabria, Basilicata, and Molise, along with Tuscany and Sardinia, experienced a very limited number of hospitalizations (fewer than 300) during this first wave. This may result in greater variability in the estimates and a lower degree of reliability for the corresponding hazard ratios. When comparing the estimated effects of other covariates to those from the full-year model, the direction of the effects remains consistent, though some variation in magnitude is observed. In this model, exposure to chronic respiratory disease increases the risk of in-hospital mortality by 79% (HR: 1.79, CI: 1.52-2.09, p < 0.001), slightly higher than the 71% increase estimated in the overall analysis. An exception is the Charlson Comorbidity Index, which, in this wave-specific model, is associated with a 5% decrease in mortality risk per unit increase in the score (HR: 0.95, CI: 0.92-0.99, p < 0.01), contrary to its effect in the general model. Another noteworthy divergence concerns the presence of dementia, which appears to be protective in this specification (HR: 0.94, CI: 0.91-0.98, p < 0.01).


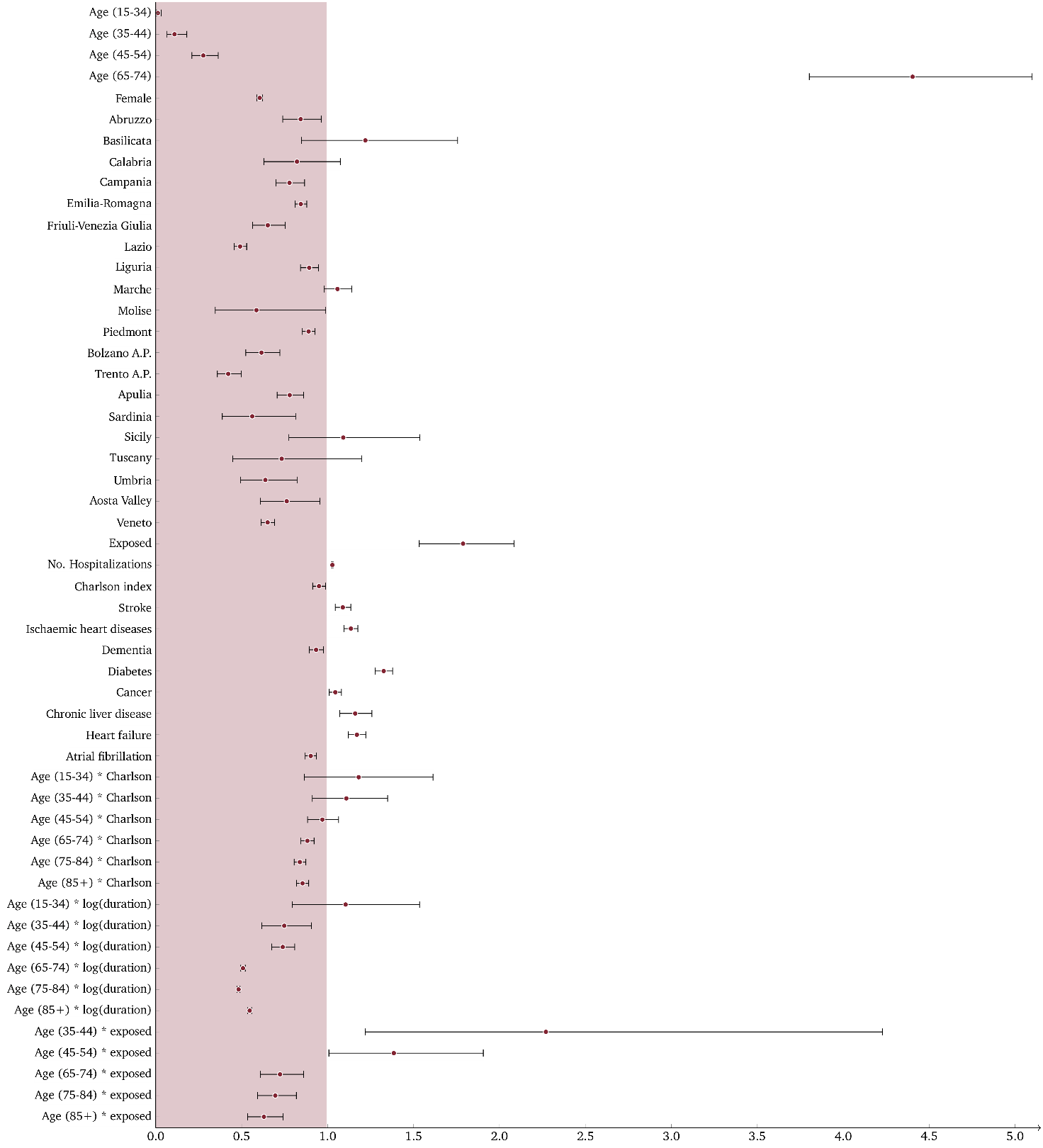
Figure 3 - Hazard ratios and 95% confidence intervals for the covariates of the Cox model restricted to the first wave of the pandemic

During the second wave of the pandemic (August 1st to December 31st, 2020), the risk of death associated with living in Lazio, Abruzzo, Sicily, Tuscany, Umbria, or Aosta Valley was not significantly different from that of Lombardy. Outside these regions, a clear north-south divide emerged: Basilicata, Calabria, Campania, Molise, and Apulia showed a higher risk of death compared to Lombardy, while Veneto, Emilia-Romagna, Friuli-Venezia Giulia, and the Autonomous Provinces of Trento and Bolzano exhibited a lower risk. Liguria stood out as an exception in the North, with an 18% higher risk of death than Lombardy (HR: 1.18, CI: 1.10-1.26, p < 0.001). These results are illustrated in Figure 4. It is important to note that, similar to the first wave, Sicily, Calabria, Basilicata, Molise, and Tuscany experienced relatively few hospitalizations during this period, which may affect the reliability of these estimates. The direction of the effects for all other covariates remained consistent with those observed in the general model.
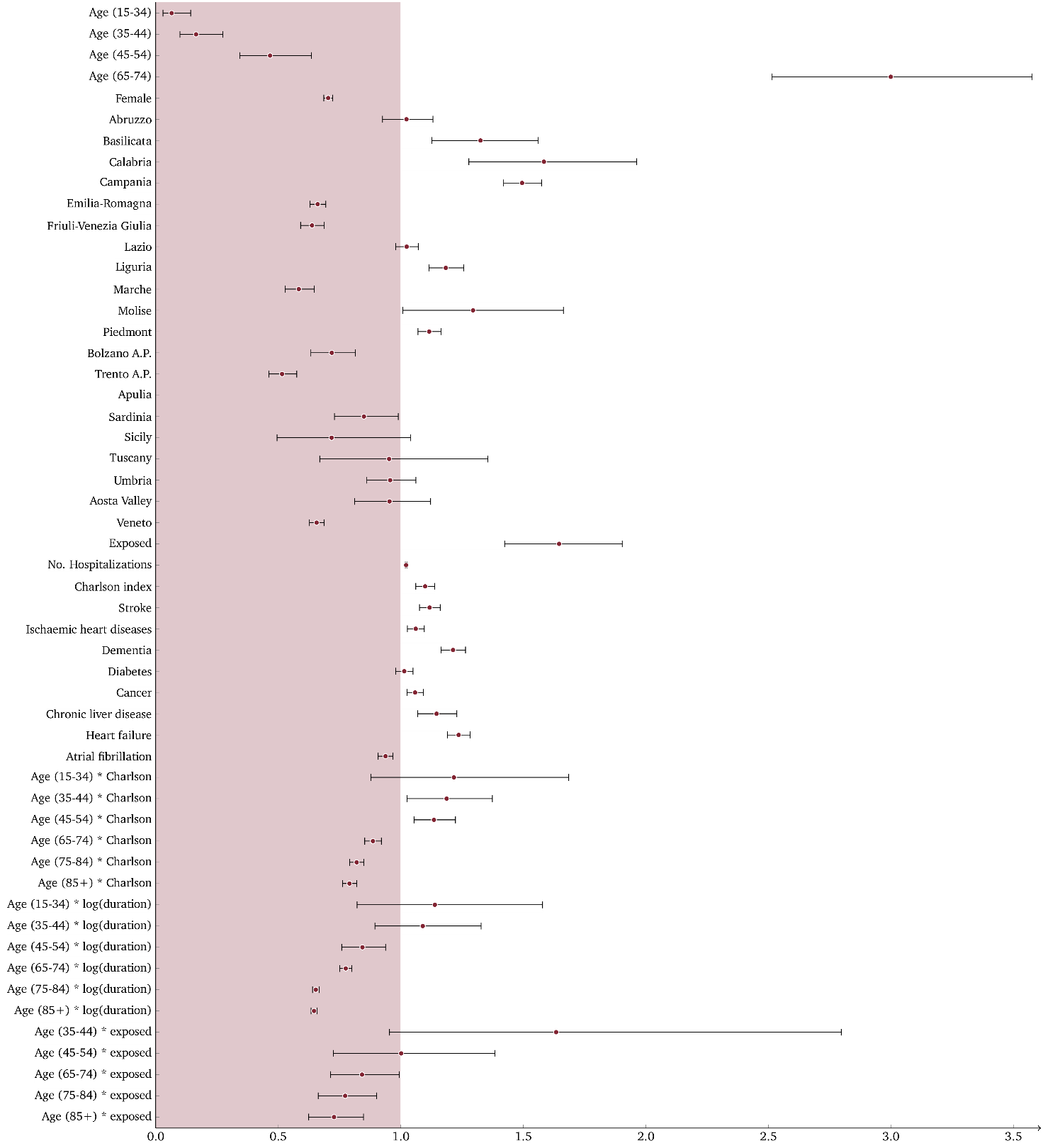


Figure 4 - Hazard ratios and 95% confidence intervals for the covariates of the Cox model restricted to the second wave of the pandemic
